# Supplementary material for: ProCS15: a DFT-based chemical shift predictor for backbone and Cβ atoms in proteins
Source: PeerJ. 2015 Oct 20;3:e1344. doi: 10.7717/peerj.1344 (PMC4662583; doi:10.7717/peerj.1344)
Supplement: Table S1 — Column 1 contains the grid spacing in the datafile. Column 2 is the size of the data files for a single atom type after data compression. Column 3 is the amount of initial generated samples. Column 4 is number of chemical shift data points after the geometry optimization and NMR calculations. Column 5 is the interpolation method used to interpolate the missing data points. Column 6 is the amino acid’s number of side chain angles in ProCS15. [file peerj-03-1344-s001.pdf]

ProCS15 Supplementary material

August 23, 2015

Table 1: Overview Table. Column 0 is the central residue type in the tripeptide. Column 1 contains the grid spacing in the datafile. Column 2 is the size of the data files for a single atom type after data compression. Column 3 is the amount of initial generated samples. Column 4 is number of chemical shift data points after the geometry optimization and NMR calculations. Column 5 is the interpolation method used to interpolate the missing data points. Column 6 is the amino acid's number of side chain angles in ProCS15.

Amino Acid	Spacing	Data file size	Samples	Data points	Interpolation	Side chain angles
Glycine	1°	3.0 MB	361	344	Cubic	0
Alanine	1°	3.0 MB	361	343	Cubic	0
Proline	1°	3.0 MB	361	246	Cubic	0
Serine	5°	9.0 MB	6859	6259	Cubic	1
Cysteine	5°	9.0 MB	6859	6326	Cubic	1
Valine	5°	9.0 MB	6859	5861	Cubic	1
Threonine	20°	3.0 MB	130321	114464	Nearest	2
Asparagine	20°	3.0 MB	130321	113566	Nearest	2
Aspartic Acid	20°	3.0 MB	130321	113790	Nearest	2
Histidine	20°	3.0 MB	130321	110787	Nearest	2
Isoleucine	20°	3.0 MB	130321	93722	Nearest	2
Leucine	20°	3.0 MB	130321	97803	Nearest	2
Phenylalanine	20°	3.0 MB	130321	107570	Nearest	2
Tryptophan	20°	3.0 MB	130321	101471	Nearest	2
Tyrosine	20°	3.0 MB	130321	111975	Nearest	2
Glutamine	20°	57.0 MB	143769	130134	Nearest	3
Glutamic Acid	20°	57.0 MB	144360	129638	Nearest	3
Methionine	20°	57.0 MB	144341	129019	Nearest	3
Arginine	20°	1.0 GB	360909	327057	Nearest	4
Lysine	20°	1.0 GB	360909	326607	Nearest	4
